# Resistance exercise training improves glucose homeostasis by enhancing insulin secretion in C57BL/6 mice

**DOI:** 10.1038/s41598-021-88105-x

**Published:** 2021-04-21

**Authors:** Gabriela Alves Bronczek, Gabriela Moreira Soares, Jaqueline Fernandes de Barros, Jean Franciesco Vettorazzi, Mirian Ayumi Kurauti, Emílio Marconato-Júnior, Lucas Zangerolamo, Carine Marmentini, Antonio Carlos Boschero, José Maria Costa-Júnior

**Affiliations:** 1 Obesity and Comorbidities Research Center, Institute of Biology, University of Campinas (UNICAMP), Campinas, São Paulo Brazil; 2 Educational Union of Cascavel—UNIVEL, Cascavel, Paraná Brazil; 3 Department of Physiological Sciences, Biological Sciences Center, State University of Maringá (UEM), Maringá, Paraná Brazil

**Keywords:** Cell biology, Molecular biology, Physiology, Systems biology, Diseases, Endocrinology

## Abstract

Resistance exercise exerts beneficial effects on glycemic control, which could be mediated by exercise-induced humoral factors released in the bloodstream. Here, we used C57Bl/6 healthy mice, submitted to resistance exercise training for 10 weeks. Trained mice presented higher muscle weight and maximum voluntary carrying capacity, combined with reduced body weight gain and fat deposition. Resistance training improved glucose tolerance and reduced glycemia, with no alterations in insulin sensitivity. In addition, trained mice displayed higher insulinemia in fed state, associated with increased glucose-stimulated insulin secretion. Islets from trained mice showed reduced expression of genes related to endoplasmic reticulum (ER) stress, associated with increased expression of Ins2. INS-1E beta-cells incubated with serum from trained mice displayed similar pattern of insulin secretion and gene expression than isolated islets from trained mice. When exposed to CPA (an ER stress inducer), the serum from trained mice partially preserved the secretory function of INS-1E cells, and prevented CPA-induced apoptosis. These data suggest that resistance training, in healthy mice, improves glucose homeostasis by enhancing insulin secretion, which could be driven, at least in part, by humoral factors.

## Introduction

Insulin resistance imposes an increase in the demand for pancreatic beta-cell to produce and secrete insulin. The maintenance of such overload for a long period triggers pancreatic beta-cell failure leading to type-2 diabetes (T2D) development^1–3^. Whereas type-1 diabetes (T1D) is characterized by autoimmune attack against pancreatic beta-cells, which leads to insulitis and, consequently, cellular death^2, 4^.

Endurance exercise is an important strategy to improve glycemic control and beta-cell function in T1D and T2D patients^5–10^. This effect could be due to an intrinsic effect of endurance exercise upon pancreatic beta-cells, and also to a mechanism dependent on some factor released into the circulation. In fact, previous studies have shown that serum from endurance-trained mice and humans reduce cellular death in a beta-cell line exposed to in vitro model of diabetes induced by pro-inflammatory cytokines or chemical endoplasmic reticulum stressors^11, 12^.

Resistance exercise has also shown beneficial effects on glycemic control and insulin secretion of diabetic patients, by reducing fasting glucose, insulin and HbA1c^13–16^; and improving beta-cell function^17, 18^. However, the majority of studies were conducted in human subjects, which unable pancreatic islet isolation for ex vivo insulin secretion analysis. Furthermore, not so far has one explored the exposition of pancreatic beta-cells to serum from resistance-trained mammals to verify potential factors released during this type of exercise that may also impact these cells.

Here, we hypothesize that resistance exercise training may improve pancreatic beta-cell function and insulin secretion, which may be associated with some factor released into the bloodstream in response to this type of exercise. To test this hypothesis, we submitted C57Bl/6 mice to resistance exercise training during 10 weeks, and demonstrated that trained mice displayed reduced glucose levels during a glucose challenge by increasing the capacity of pancreatic beta-cells to secrete insulin. Moreover, the serum from trained mice reduced beta-cell injury and apoptosis in INS-1E cells exposed to the chemical ER stressor CPA.

Our data indicate that resistance exercise could be an interesting approach to improve beta-cell functions, and that humoral factors present in the bloodstream, after performing this type of exercise, may be associated with this process. Moreover, it may be an alternative exercise regimen for individuals with difficulties to perform endurance exercise, such as morbid obese, elderly individuals and diabetic foot syndrome patients.

## Results

### Resistance exercise training induces adaptation in mice

**Figure 1:**
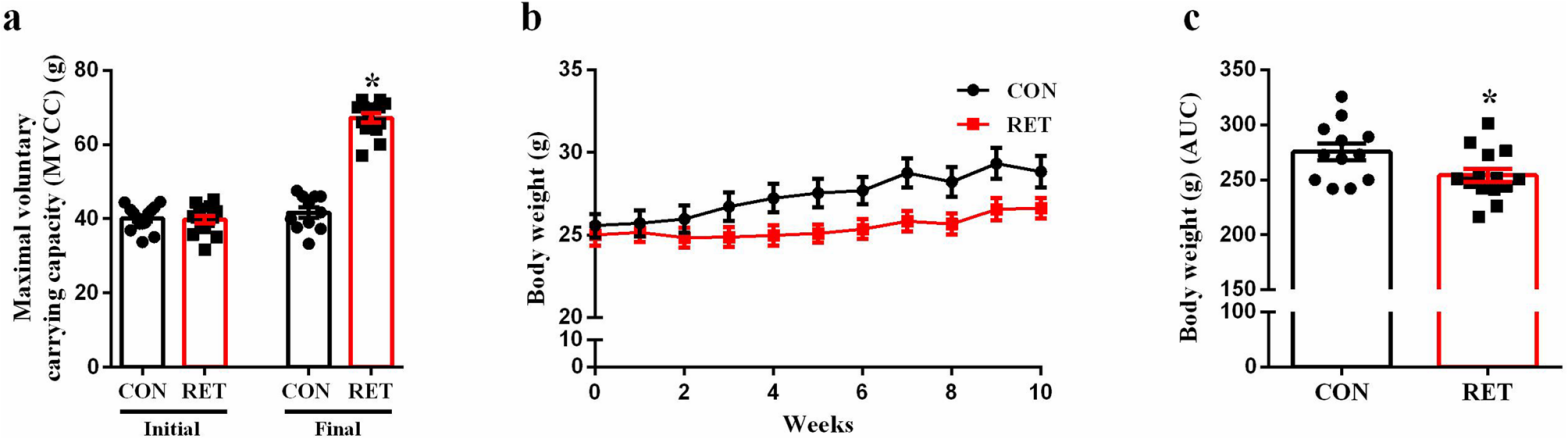
Resistance exercise increases maximal voluntary carrying capacity (MVCC) and reduces weight gain in trained mice. Initial and final maximal voluntary carrying capacity (MVCC) of CON (black bar, n = 12) and RET (red bar, n = 14) (**a**). Body weight over the course of 10 weeks (**b**) and its respective area under the curve (AUC) (**c**) of CON (black bar, n = 12) and RET (red bar, n = 14). Data are the mean ± SEM. *P ≤ 0.05 (Student’s t-test).

Firstly, we evaluated the maximum voluntary carrying capacity (MVCC) to assess the efficiency of our training program. As expected, after 10 weeks of training, resistance exercise training (RET) mice presented higher MVCC, compared with control (CON) mice (p < 0.0001, Fig. 1A). Also, the training protocol increased the performance as judged by the ability of trained mice to carry progressively heavier loads, in the course of the 10-week period (see Supplementary Fig. S1 online). Moreover, resistance training reduced body weight gain (p = 0.0384, Fig. 1B,C). RET mice also presented reduced perigonadal (p < 0.0001) and retroperitoneal (p = 0.0003) fat pads, as well as, increased gastrocnemius (p = 0.0093) and soleus (p = 0.0021) weight (Table 1).

**Table 1:** Final characterization of Control (CON) and Resistance exercise training (RET) mice.

	CON	RET	p-value
Retroperitoneal fat pad (% body weight) (g)	214.3 ± 15.59	129.3 ± 10.47*	0.0003
Perigonadal fat pad (% body weight) (g)	925.0 ± 84.95	533.0 ± 27.63*	< 0.0001
Gastrocnemius (% body weight) (g)	928.8 ± 25.41	1021 ± 19.13*	0.0093
Soleus (% body weight) (g)	150.1 ± 6.43	184.9 ± 6.80*	0.0021

(*) Indicates statistic differences between groups. Data are presented as the mean ± SEM, n = 10. Student’s t-test or Mann–Whitney test.

### Resistance training modulates glucose metabolism

**Figure 2:**
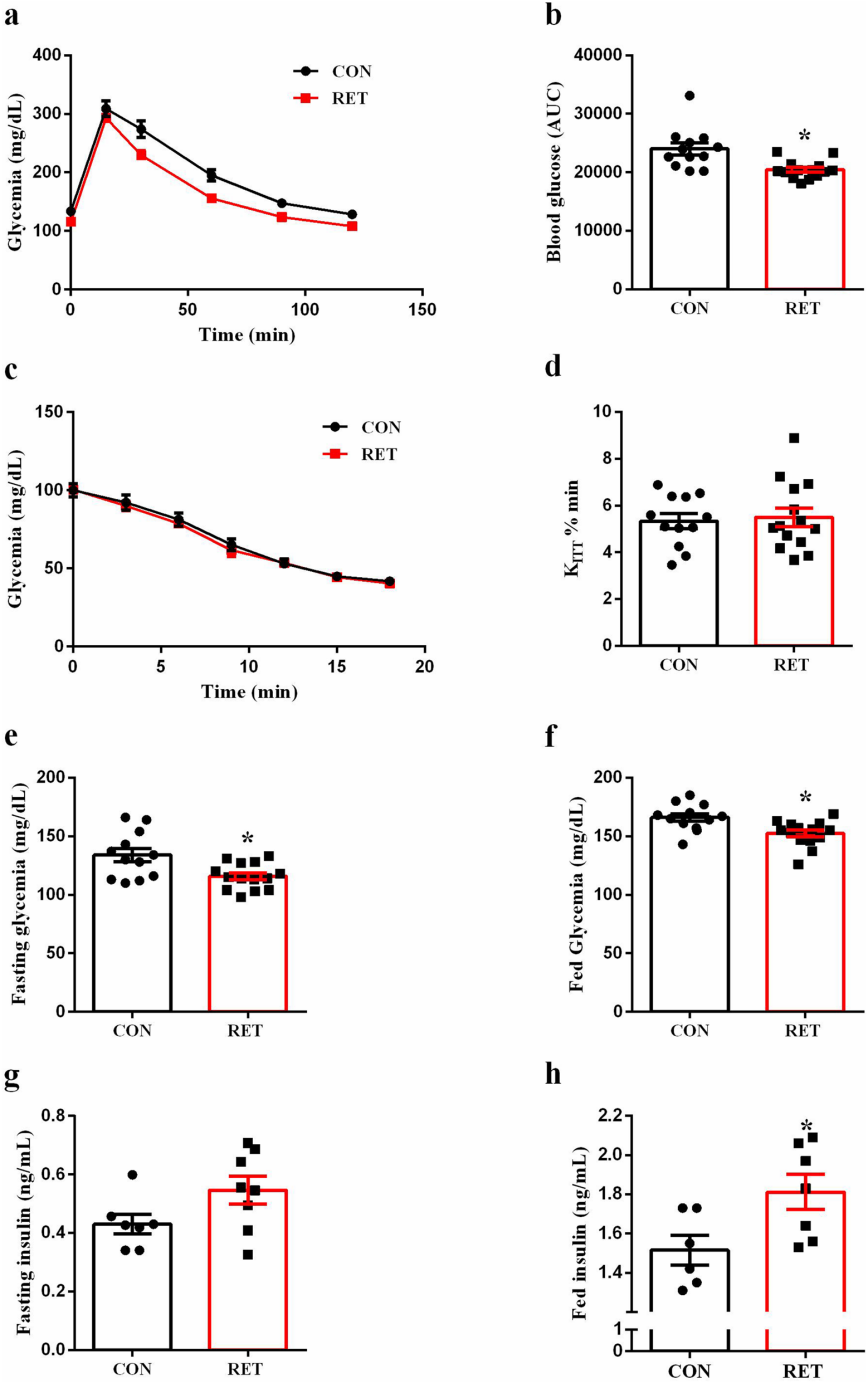
Resistance training improves glucose tolerance, reduces glycemia and increases fed insulinemia. Blood glucose of CON (black bar, n = 12) and RET (red bar, n = 14) during ipGTT (**a**). Area under the curve (AUC) of total blood glucose concentration of CON (black bar, n = 12) and RET (red bar, n = 14) during ipGTT (**b**). Blood glucose of CON (black bar, n = 12) and RET (red bar, n = 14) during ipITT (**c**). Constant of glucose disappearance expressed by K_ITT_, CON (black bar, n = 12) and RET (red bar, n = 14) (**d**). Fasting (**e**) and fed glycemia (**f**) of CON (black bar, n = 12) and RET (red bar, n = 14). Plasma insulin of CON (black bar, n = 6–7) and RET (red bar, n = 7–8) in fasting (**g**) and fed (**h**) states. Data are the mean ± SEM. *P ≤ 0.05 (Student’s t-test).

We performed intraperitoneal glucose and insulin tolerance tests (ipGTT and ipITT) to access the effects of resistance exercise training on glucose homeostasis. After glucose administration, both groups had a maximal glucose peak at 15–30 min (Fig. 2A). However, RET mice displayed improved glucose tolerance (Fig. 2A), as determined by the lower AUC of blood glucose during ipGTT (p = 0.0021, Fig. 2B). Moreover, there was no difference between groups regarding the insulin sensitivity, as judged by the constant of glucose disappearance expressed by K_ITT_ (Fig. 2C,D). Fasting (p = 0.0073, Fig. 2E) and fed (p = 0.0053, Fig. 2F) glycemia were also evaluated and trained mice presented reduced glycemia in both states. Finally, we observed that plasma insulin levels in fed (p = 0.0307, Fig. 2H), but not fasted (Fig. 2G) state, was significantly higher in the RET group, which may contribute to reduced glycemia as well as improved glucose tolerance in this group.

### Resistance exercise training increases glucose-stimulated insulin secretion and improves beta-cell function

**Figure 3:**
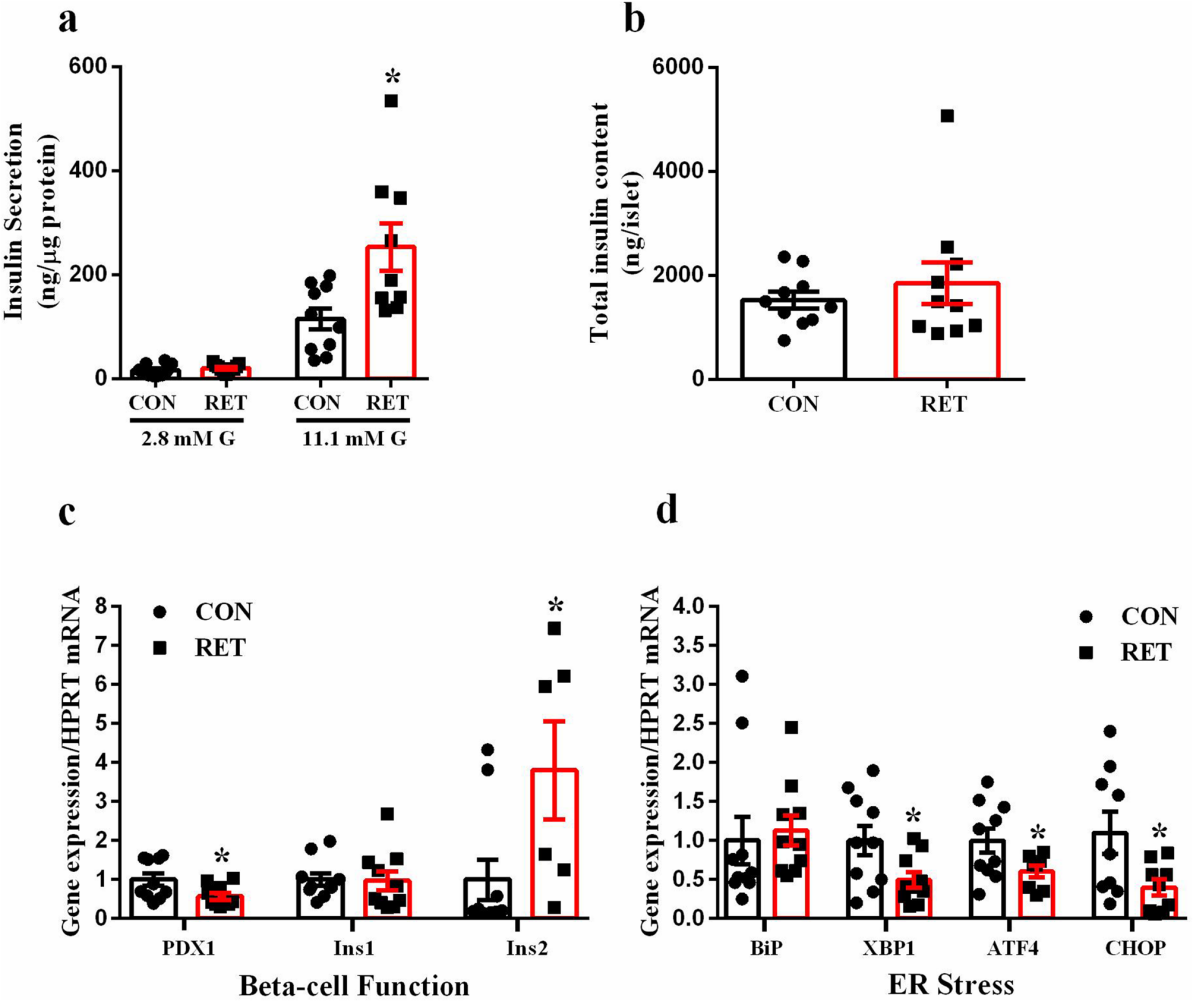
Resistance training increases glucose-stimulated insulin secretion and improves beta-cell function. Glucose-stimulated insulin secretion (**a**) and total insulin content (**b**) of islets from CON (black bar, n = 10) and RET (red bar, n = 9–11). Real-time PCR assay of PDX1, Ins1 and Ins2 mRNA levels (**c**) in pancreatic islets of CON (black bar, n = 10) and RET (red bar, n = 6–10). Real-time PCR assay of BiP, XBP1, ATF4 and CHOP mRNA levels (**d**) in pancreatic islets of CON (black bar, n = 9–10) and RET (red bar, n = 8–10). The relative expression of mRNAs was determined after normalization with HPRT. Data are the mean ± SEM. *P ≤ 0.05 (Student’s t-test).

To investigate whether the elevated plasma insulin concentration in the RET mice in fed state could be related to beta-cell function or not, we assessed glucose-stimulated insulin secretion (GSIS) from isolated pancreatic islets. Islets from trained mice released more insulin than control mice, only at high glucose concentration (p = 0.0106, Fig. 3A). However, there was no difference between groups regarding the total insulin content (Fig. 3B). Therefore, the improvement in glucose homeostasis, observed in RET mice, may be explained by an increase in GSIS rather than an alteration in insulin sensitivity. Interestingly, there was a reduction in the expression of some genes related to ER stress, such as XBP1 (p = 0.0275), ATF4 (p = 0.0469) and CHOP (p = 0.0290) (Fig. 3D), as well as, an increase in the expression of the Ins2 gene (p = 0.0302, Fig. 3C), in pancreatic islets from trained mice. This could contribute, at least in part, to the improvement in insulin secretion observed in the pancreatic islet of these mice. Paradoxically, the mRNA expression of PDX1 (p = 0.0274, Fig. 3C), a gene related to pancreatic beta-cell proliferation and survival, was reduced in RET mice, compared to controls.

### Serum from resistance-trained mice protects INS-1E beta-cells from ER stress injury and apoptosis

**Figure 4:**
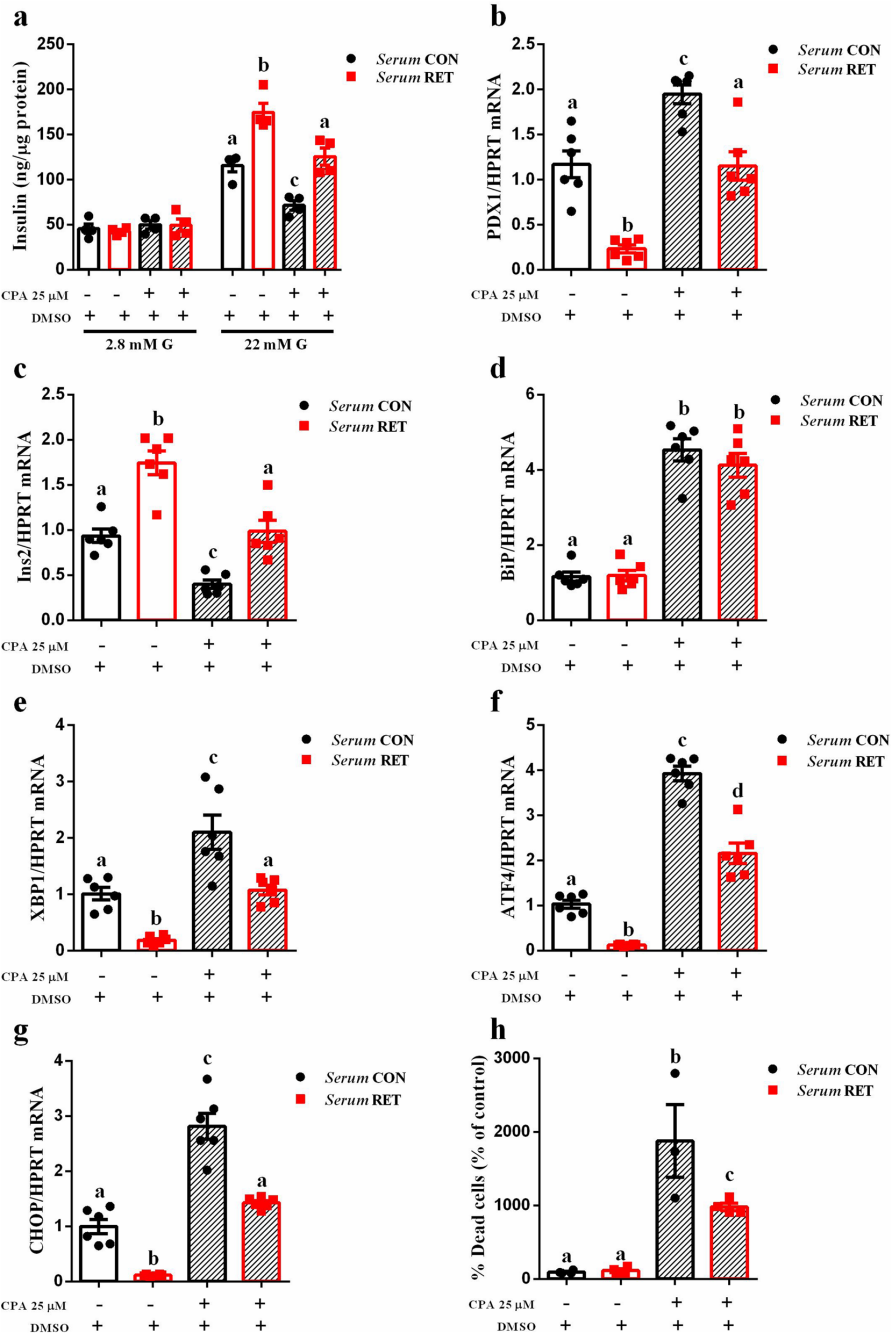
Serum from resistance-trained mice protects INS-1E cells from ER stress injury and apoptosis. INS-1E cells were incubated with conditioned medium with 10% of serum from control (striped black bar) or trained mice (striped red bar) for 24 h, followed by 16 h exposure to 25 µM CPA. Insulin secretion from INS1-E cells exposed to 2.8 or 22.2 mM glucose (n = 4) (**a**), Real-time PCR assay of PDX1 (**b**), Ins2 (**c**), BiP (**d**), XBP1 (**e**), ATF4 (**f**) and CHOP (**g**) mRNA levels (n = 6) in INS-1E cells from different treatments as indicated in the graph. The relative expression of mRNAs was determined after normalization with HPRT. Cell apoptosis was measured by HO and PI staining (n = 3–4). The % of dead cells (HO/PI stained cells ratio) is represented as % of control (Serum CON cells without CPA) (**h**). Data are the mean ± SEM. Different letters indicate statistic differences between groups, P ≤ 0.05 (One-Way ANOVA).

Based on our preceding findings, we hypothesized that the alterations in pancreatic islets from trained mice were mediated by exercise-induced factors secreted in the bloodstream during resistance exercise. To test this hypothesis, a rat pancreatic beta-cell line (INS-1E) was incubated with medium containing 10% of serum from control or trained mice for 24 h; followed by exposure to cyclopiazonic acid (CPA), an inductor of ER stress, for 16 h. Interestingly, under normal conditions (DMSO) INS-1E cells incubated with trained serum secrete more insulin than cells incubated with serum from control mice, in response to high glucose concentrations (Serum CON *vs*. Serum RET p = 0.0015, Fig. 4A), similar to the outcomes observed in the ex vivo conditions (Fig. 3A). Furthermore, when cells were exposed to CPA, insulin secretion was impaired in cells previously incubated with control serum, whereas the serum from trained mice partially preserved the secretory function of these cells (Serum CON *vs*. Serum RET p = 0.0029, Fig. 4A).

When we analyzed the expression of various genes related to beta-cell function and ER stress, we observed that INS-1E cells incubated in normal conditions (DMSO), but in the presence of serum from trained mice, presented: reduced expression of PDX1 (Serum CON *vs.* Serum RET p = 0.0001); increased expression of Ins2 (Serum CON *vs.* Serum RET p < 0.0001); as well as reduced expression of ER stress markers XBP1, ATF4 and CHOP (Serum CON *vs.* Serum RET p = 0.0117, p = 0.0013, p = 0.0008, respectively) (Fig. 4B–G). These results are in line with data obtained with pancreatic islets from trained mice (Fig. 3C,D). Moreover, CPA treatment reduced expression of Ins2, increased expression of PDX1 and ER stress markers Bip, XBP1, ATF4 and CHOP (Serum CON with *vs.* without CPA p = 0.0056, p = 0.0011, p < 0.0001, p = 0.0010, p < 0.0001, p < 0.0001, respectively) in INS-1E cells, previously incubated with serum from control mice. Interestingly, INS-1E cells pre-treated with serum from trained mice and exposed to CPA presented improved beta-cell function, which was associated with increased Ins2 expression (Serum CON *vs.* Serum RET p = 0.0024) and reduced expression of ER stress markers (Serum CON *vs.* Serum RET: XBP1 p = 0.0018, ATF4 p < 0.0001, CHOP p < 0.0001) (Fig. 4B–G). Finally, in normal conditions (DMSO) there was no difference between groups (Serum CON *vs.* Serum RET) regarding apoptosis rate (Fig. 4H). Additionally, CPA treatment increased beta-cell apoptosis as measured by the fluorescent DNA-binding dyes HO and PI (Serum CON with *vs.* without CPA p = 0.0003; Serum RET with *vs.* without CPA p = 0.0306, Fig. 4H). However, INS-1E cells cultured in medium containing serum from trained mice were partially protected against CPA-induced apoptosis (Serum CON *vs.* Serum RET p = 0.0376). Taken together, these data suggest that resistance training is able to improve beta-cell function, providing resistance to subsequent stresses.

## Discussion

Here, we provide evidence to suggest that resistance training improves glucose tolerance and reduces fed and fasting state glycemia. These results are probably due to increased insulin secretion and reduced ER stress, which we observed both in pancreatic islet from trained mice and INS-1E beta-cells incubated with serum from these mice. Based on these findings, we propose that the improvement induced by resistance training on glucose tolerance involves an increment of GSIS that could be driven, at least in part, by humoral factors.

We analyzed the effects of resistance training on glucose homeostasis in mice by using glucose and insulin tolerance tests, which were performed immediately after the exercise session, 6 h and 24 h post-exercise. Curiously, we found different responses depending on the time of analysis (see Supplementary Fig. S2 online). 6 h post-exercise trained mice showed improved glucose tolerance (Fig. 2A,B) with no alterations in insulin sensitivity (Fig. 2C,D), indicating that resistance exercise may increase GSIS at this time point. All the experiments in vivo were performed 6 h post-exercise in order to explore the mechanisms underlying the increase of beta-cell function on trained mice.

In fact, plasma insulin levels were higher in trained mice in fed state (Fig. 2H), which might contribute to the reduced glycemia observed in this group (Fig. 2F). These results differ from previous findings, demonstrating that glycemic reduction induced by resistance exercise occurs by improving insulin sensitivity associated with reduced insulinemia. However, these studies were conducted in obese/overweight or diabetic humans^13–15, 18–20^ and rodents^21, 22^ which may explain these discrepancies.

Fasting glycemia was also reduced in trained mice (Fig. 2E), not explained either by insulin levels (Fig. 2G) or insulin sensitivity (Fig. 2C,D). We speculate that a slight increase in liver insulin action, which is probably below of the capacity of detection by regular insulin tolerance test, could be acting here, reducing hepatic glucose production (HGP), thus contributing to the reduced glycemia. In fact, resistance exercise increases liver insulin sensitivity in obese mice improving the control of HGP^23^.

The increase in insulinemia in fed state complies with the increase in insulin secretion, observed in isolated islets from trained mice, only at high glucose concentrations (Fig. 3A). These data indicate that insulin secretion rather than insulin action may be the key factor to improve glucose homeostasis in our experimental exercise model. In agreement with our findings, isolated pancreatic islets from rats, submitted to acute resistance exercise, secrete more insulin when exposed to high glucose concentrations and arginine^24^, demonstrating that resistance exercise is able to turn the beta-cell more efficient to secrete insulin, in response to a glucose stimulus.

In T2D individuals, resistance training also improves beta-cell function (HOMA-β)^17, 18^ but no molecular analysis has been done yet. In this way, we evaluated the expression of genes related to beta-cell function and observed that Ins2 expression was higher in islets from trained mice (Fig. 3C). Since this gene is responsible for encoding preproinsulin^25^, this data may explain, at least in part, the increase in insulinemia and insulin secretion observed in these mice.

At the same time, there was a reduction in PDX1 expression (Fig. 3C), at first glance paradoxical, since it is known that PDX1 is crucial for pancreas development, beta-cell function, replication and maintenance^26–29^. However, PDX1 forms complexes with ATF4, and the later controls the expression of genes involved with pro- or anti-apoptotic responses, depending on the type of cell stress^30^. Therefore, PDX1 could be considered as a stress sensor, and its reduced expression in islets from trained mice may be a sign of reduced stress and enhanced functioning of the insulin secreting cells. In fact, there was a reduction in the expression of genes related to ER stress (Fig. 3D) in islets from trained mice. Of note, it was demonstrated that regular exercise reverses ER dysfunctions, and muscle contraction is directly involved in UPR activation, mainly in skeletal muscle, liver and adipose tissue^31–34^.

The reduction of ER stress in pancreatic beta-cells, induced by exercise, could reflect a phenomenon known as hormesis, which is the capacity of the organism to adapt to a moderate stress, i.e. physical exercise, increasing its resistance to subsequent harmful stress^12, 35, 36^. This phenomenon helps to explain the reduction of ER stress in healthy exercised mice.

In agreement with it, we showed that the serum from resistance-trained mice was able to partially preserve insulin secretion (Fig. 4A), reduced expression of ER stress markers (Fig. 4B–G) and apoptosis rate (Fig. 4H) in INS-1E cells exposed to CPA. The serum-treated INS-1E cells (under normal conditions, without CPA) also reproduced the same outcomes observed in ex vivo isolated pancreatic islet from trained mice (Figs. 3A–D and 4A–H).

In this way, we demonstrated that resistance training in healthy mice is able to modulate UPR components in pancreatic islets and INS-1E beta-cells, leading to reduced ER stress. Sustained ER stress in beta-cells is associated with apoptosis in T1D and T2D^37^. ER stress is also related to the activation of inflammatory pathways in T1D, which can occur before the development of insulitis^38^. In addition, ER stress predisposes beta-cells to autoreactive T cell recognition^39^ contributing to the autoimmune attack. Thinking of that, therapeutic strategies that aim to reduce/alleviate ER stress and improve pancreatic beta-cell functions could benefit both patients with T1D and T2D.

Our findings also suggest a possible role of factors released in the bloodstream during resistance exercise. Similar results have been found in endurance-trained mice^11, 12^. However, further studies are needed to address if resistance exercise release specific modulators of pancreatic beta-cell into the circulation.

In fact, conditioned medium from white-glycolytic muscle cells (which are predominantly stimulated by resistance exercise) was more efficient than conditioned medium from red-oxidative muscle cells (which are predominantly stimulated by endurance exercise) to preserve insulin secretion, proliferation and survival, as well as reduced beta-cell death under pro-inflammatory conditions^40, 41^. Besides, several muscle-derived factors released in response to exercise might act upon beta-cell function and survival^42^, such as: IL-6^11^, irisin^43^, BnDF^44^, GDF-15^45^, myostatin, follistatin^46^, angiogenin and osteoprotegerin^40^.

In addition to skeletal muscle, other tissues release factors and hormones in response to exercise that may also impact beta-cells^47, 48^. For instance, IGF-1 a hormone released by the liver and also by mechanical-stretch mechanisms in local tissues^49^ is increased with exercise, especially resistance exercise^50, 51^. Taking this into account, the discovery of which molecules are involved with the effects of resistance training on glucose homeostasis may reveal new treatments for diabetes and related diseases.

In conclusion, the present study shows that resistance exercise training in healthy mice improves glucose homeostasis by enhancing insulin secretion. These effects are probably associated with factors released in the serum that may play a role in attenuating ER stress and favoring beta-cell survival. Further investigations are needed to identify the exercise-induced factors involved with this phenomenon. Collectively, our outcomes support the use of lifestyle interventions, such as resistance exercise, to both improve insulin secretion and prevent beta-cell loss in the face of harmful conditions, as in diabetes.

## Material and methods

### Experimental animals

All experiments were approved by the Animal Care Committee at UNICAMP (License Number: 5068-1) and performed in accordance with relevant named guidelines and regulations. The study was carried out in compliance with the ARRIVE guidelines. Male, 8-week-old C57Bl/6 mice were obtained from the breeding colony at UNICAMP and maintained at 22 ± 1 °C on a 12 h light–dark cycle. During the experimental period, mice had free access to water and chow diet. The animals were divided into two groups: the control (CON) group, which remained sedentary throughout the experimental period, and the resistance exercise training (RET) group, which underwent resistance exercise training during 10 weeks. All animals were housed collectively (5 animals per cage) and weighted once a week. Also, fasting and fed blood glucose was measured on the 9th week of training. At the end of the training program, mice were euthanized for blood collection, by decapitation after inhalation of isoflurane. The muscles soleus and gastrocnemius, as well as perigonadal and retroperitoneal fat pads were removed and weighted. All the experiments were performed 6 h after the exercise session.

### Description of apparatus for performing resistance exercise training for mice

To perform the resistance exercise protocol, a ladder of iron feet and stainless steel steps was used. The ladder is 5 cm wide, 105 cm high with 1 cm distance between the steps, and an angle of 80 degrees to the ground (AVS PROJECTS, São Carlos, Brazil). Thus, the animals performed 8–12 dynamic movements. There is a housing chamber (9 × 9 × 9 cm) at the top of the ladder, in which the animals are able to rest between each climb. The load apparatus consisted in a conical plastic tube with approximately 5 cm of height and 2.5 cm of diameter, and it was fixed across the length of the animal’s tail using an adhesive tape^23, 52^.

### Maximum voluntary carrying capacity (MVCC) determination and resistance exercise training protocol

Resistance exercise training was performed according to the animal model described by Hornberger and Farrar^52^ with some modifications. Firstly, RET mice were familiarized with climbing the ladder with a load apparatus secured to the proximal portion of the tail. At the top of the ladder, the mice reached the housing chamber and were allowed to rest between climbs. To familiarize with the exercise protocol, the mice were kept in the housing chamber for 120 s before each attempt to climb. Three attempts were performed per day and the mice were encouraged to climb 35, 55 and 70 cm until they reached the housing chamber. Mice were encouraged to climb the ladder by touching their tails. This protocol was performed over four consecutive days. After this period, we determined the maximum voluntary carrying capacity (MVCC) of each animal^23, 52, 53^, before the beginning of the training program. The test for MVCC determination was initiated with a climb carrying a load of 75% of the animal’s body weight and upon successful completion; an incremental load of 10% of the animal’s body weight was added to the load apparatus. This procedure was successively repeated (with a rest interval of 120 s between climbs) until a load was reached with which the animal could not climb the entire length of the ladder. Failure was defined as the inability to climb the ladder following three successive tail stimuli. The highest load successfully carried was considered the MVCC of the mice. Based on the MVCC test, the training sessions consisted of 8 climbs at four different loads (two climbs with each load): 50%, 75%, 90% and 100% of the animal’s MVCC, with a rest interval of 60 s between climbs. The resistance training was performed 5 days per week with 2 days of rest, during a 10-week period. During this period, MVCC was determined once a week (on Fridays) to set the appropriate load for each animal. CON mice remained in cage during the training period. These animals were only exposed to the training ladder at week 0 (initial) and 10 (final). At this two time points, CON mice were submitted to the adaptation protocol, as described above, and then performed the MVCC test.

### Intraperitoneal glucose (ipGTT) and insulin (ipITT) tolerance tests

On the 8th week of the training program, mice were subjected to 6 h fasting after the training session, to perform the ipGTT. The fasting blood glucose level was measured (time 0) by a glucometer. After, the mice received an i.p. glucose dose of 2 g/kg, and glycemia was measured at 15, 30, 60, 90, and 120 min. Two days later, after the training session, mice were subjected to 6 h fasting for the ipITT, and the glycemia was measured before (time 0) and 3, 6, 9, 12, 15 and 18 min after the i.p. administration of 1 U/kg insulin. To assess the decay rate constant (K_ITT_), glycemia was converted to natural logarithmic values (Ln). The slope was calculated using linear regression (time × Ln ^glycemia^) and we obtained the glycemia decay rate constant (% min^−1^) by multiplying the result by 100^54^.

### Plasma insulin measurement

For insulin measurements, blood samples were collected in fed and fasted states at the end of the training program. To obtain plasma samples, blood samples were centrifuged at 11,000 rpm, for 15 min, 4 °C. To measure plasma insulin, Mouse insulin Elisa Kit (Darmstadt, Germany, cat. #EZRMI-13 K) was used, and the assay was performed as indicated by the kit protocol.

### Islet isolation, insulin secretion and insulin content

For static insulin secretion, islets were isolated by collagenase digestion of the pancreas. In brief, 6 h after the last exercise session, mice were euthanized and the pancreas was inflated with Hanks balanced salt solution containing 3 mg collagenase/ml, excised and then continuously shaken at 37 °C for 18 min. Then, islets were selected with a micropipette under a microscope to exclude any contaminating tissues^55, 56^. Groups of 5 pancreatic islets were incubated for 30 min with Krebs-bicarbonate buffer (KBB; (in mmol/l) 115 NaCl, 5 KCl, 2.56 CaCl_2_, 1 MgCl_2_, 10 NaHCO_3_, 15 HEPES), supplemented with 5.6 mmol/l glucose and 0.3% bovine serum albumin (BSA) and equilibrated with mixture of 95% O_2_/5% CO_2_ to regulate the pH at 7.4, and at the temperature of 37 °C. Next, the medium was removed and immediately replaced with fresh KBB medium containing 2.8 (low concentration) or 11.1(high concentration) mM glucose. After 1 h of incubation time, the medium was removed and stored at − 20 °C. For islet insulin and protein content, the groups of five islets were collected and transferred to tubes containing 20 µl of alcohol/acid solution. Insulin levels and insulin content were measured by a radioimmunoassay (RIA)^57^. Total protein was measured by Bradford^58^. Insulin secretion was normalized by total protein.

### INS-1E cell culture and treatment

Rat insulin-producing INS-1E cells (a kind gift from Professor C. Wollheim, Centre Medical Universitaire, Geneva, Switzerland) were cultured in RPMI 1640 medium (VITROCELL, SP, Brazil) and supplemented with 5% v/v of fetal bovine serum (FBS, VITROCELL, SP, Brazil), HEPES 10 mmol/l, sodium pyruvate 1 mmol/l and 2-mercaptoethanol 50 µmol/l with 11 mmol/l glucose; in a humidified atmosphere at 37 °C and 5% CO_2_. Cells were used at passages 60–70. INS-1E cells were seeded in 24-well, 48-well or 96-well culture plates until 70–80% confluence. Then, cells were incubated with medium containing 10% of serum from control or trained mice (without FBS), for 24 h. Next, the medium was replaced by fresh growth medium (with FBS) containing dimethyl sulfoxide (DMSO) or 25 µM of cyclopiazonic acid (CPA) for 16 h^12^. Finally, the cells were washed with phosphate buffered saline and used for insulin secretion, apoptosis measurement (by HO-PI fluorescence quantification) and Real-Time PCR assays.

### Insulin secretion in INS-1E cells

After the culture treatment period, INS-1E cells (seeded in 24-well culture plates) were incubated for 1 h at 37 °C in KBB without glucose. This solution was replaced with fresh KBB containing 2.8 (low concentration) or 22.2 (high concentration) mM glucose for 1 h. At the end of the incubation period, supernatants were collected and stored at − 20 °C until insulin was measured by an Elisa Kit (Darmstadt, Germany, cat. #EZRMI-13K). INS-1E cells were subsequently washed with PBS, lysed in 60µL of urea/thiourea buffer (7 M urea, 2 M thiourea, 100 mM Tris pH 7.5, 10 mM sodium pyrophosphate, 100 mM sodium fluoride, 10 mM ethylenediaminetetraacetic acid (EDTA), 10 mM sodium orthovanadate, 2 mM phenylmethylsulfonyl fluoride (PMSF), 1% Triton X-100 and 0.1 mg/mL aprotinin, 4 °C) and stored at -20ºC until assayed for total protein measurement by Bradford^58^. Insulin secretion was normalized by total protein.

### HO-PI fluorescence quantification

The percentage of dead and viable cells was assessed using the fluorescence quantification of DNA-binding dyes propidium iodide (PI; 5 µg/ml) and Hoechst 33342 (HO; 10 µg/ml) (both SIGMA-ALDRICH), respectively. After serum and CPA treatment, INS-1E cells (seeded in 96-well culture plates) were incubated with HO-PI for 15 min. Next, the cells were observed using a *High-Content Imaging System* (ImageXpress Micro Confocal, MOLECULAR DEVICES) and the module *Live and Dead* in the *MetaXpress* software (MOLECULAR DEVICES). The % of dead cells (which was calculated as the ratio of HO/PI stained cells) is represented as % of control (Serum CON cells without CPA).

### mRNA isolation and real time quantitative PCR

The extraction of total RNA content of the islet (n = 10) and INS-1E cells (seeded in 48-well culture plates, n = 6) was performed using TRIzol reagent (GIBCO BRL, Life Technologies), following phenol–chloroform RNA extraction, according to the manufacturer’s recommendations. Nanodrop (NANODROP THERMO SCIENTIFIC, Wilmington, DE, USA) was used to measure RNA concentration. cDNA was prepared using 1 µg RNA for islets and 0.3 µg RNA for INS-1E cells and MultiScribe reverse transcriptase (APPLIED BIOSYSTEMS, Foster City, CA, USA). For PCR reactions, we used SYBR-green master mix (APPLIED BIOSYSTEMS, Foster City, CA, USA). 7500 Fast Real-time PCR System (APPLIED BIOSYSTEMS, Foster City, CA, USA) was used for quantification. The specificities of amplifications were verified by melting-curve analyses. The relative expression of mRNAs was determined after normalization with HPRT, using the 2^−ΔΔCt^ method^59^. Primer sequences used for real-time PCR assays of pancreatic islets were as follows: PDX1 forward: 5′-attcttgagggcacgagagc-3′; PDX1 reverse: 5′-agctcagggctgtttttcca-3′; Ins1 forward: 5′-gccaaacagcaaagtccagg-3′, Ins1 reverse: 5′-gttgaaacaatgacctgcttgc-3′; Ins2 forward: 5′-gtcaagcagcacctttgtgg-3′; Ins2 reverse: 5′-cagttgtgccacttgtgggt-3′; Bip forward: 5′-acttggggaccacctattcct-3′, Bip reverse: 5′-atcgccaatcagacgctcc-3′; XBP1 forward: 5′-agcagcaagtggtggatttg-3′, XBP1 reverse: 5′-gagttttctcccgtaaaagctga-3′; ATF4 forward: 5′-ggacgatctctaacgccaca-3′, ATF4 reverse: 5′-cttgtcgctggagaacccat-3′; CHOP forward: 5′-ctggaagcctggtatgaggat-3′, CHOP reverse: 5′-cagggtcaagagtagtgaaggt-3′; HPRT forward: 5′-tcagtcaacgggggacataaa-3′, HPRT reverse: 5′-ggggctgtactgcttaaccag-3′. Primer sequences used for real-time PCR assays of INS-1E cells were as follows: PDX1 forward: 5′-gtagtagcgggacaacgagc-3′; PDX1 reverse: 5′-cgaggttacggcacaatcc-3′; Ins2 forward: 5′-atcctctgggagccccgc-3′; Ins2 reverse: 5′-agagagcttccaccaag-3′; Bip forward: 5′-acttggggaccacctattcct-3′, Bip reverse: 5′-atcgccaatcagacgctcc-3′; XBP1 forward: 5′-gagtccgcagcaggtg-3′, XBP1 reverse: 5′- gcgtcagaatccatggga-3′; ATF4 forward: 5′-gttggtcagtgcctcagaca-3′, ATF4 reverse: 5′-cattcgaaacagagcatcga-3′; CHOP forward: 5′-ctggaagcctggtatgaggat-3′, CHOP reverse: 5′-cagggtcaagagtagtgaaggt-3′; HPRT forward: 5′-tcctcatggactgattatggaca-3′, HPRT reverse: 5′-taatccagcaggtcagcaaaga-3′.

### Statistical analysis

The data are presented as the mean ± standard error of the mean (SEM). The sample size (n) used for the statistical analysis of each group in the experiments is described in the figure legends. To evaluate data normality, we applied the Shapiro–Wilk test. When normal, we used the parametric Student’s t-test (to compare two groups) or One-Way ANOVA with an unpaired Tukey’s post-hoc test (to compare 3 + groups); otherwise, the non-parametric Mann–Whitney test (to compare two groups) was adopted. The difference between groups was considered statistically significant if *p* ≤ 0.05.

## Supplementary Information

Supplementary Information

## Data Availability

All data generated or analyzed during this study are included in this published article (and its Supplementary Information files).
