# Supplementary material for: Resistance exercise training improves glucose homeostasis by enhancing insulin secretion in C57BL/6 mice
Source: Sci Rep. 2021 Apr 21;11:8574. doi: 10.1038/s41598-021-88105-x (PMC8060292; doi:10.1038/s41598-021-88105-x)

Resistance exercise training improves glucose homeostasis by enhancing insulin secretion in C57BL/6 mice

Gabriela Alves Bronczek[1], Gabriela Moreira Soares[1], Jaqueline Fernandes de Barros[1], Jean Franciesco Vettorazzi[2], Mirian Ayumi Kurauti[3], Emílio Marconato-Júnior[1], Lucas Zangerolamo[1], Carine Marmentini[1], Antonio Carlos Boschero[1], José Maria Costa-Júnior[1*].

[1]Obesity and Comorbidities Research Center, Institute of Biology, University of Campinas (UNICAMP), Campinas, São Paulo, Brazil

[2]Educational Union of Cascavel – UNIVEL, Cascavel, Paraná, Brazil

[3]Department of Physiological Sciences, Biological Sciences Center, State University of Maringá (UEM), Maringá, Paraná, Brazil

* Correspondence:

Dr José Maria Costa Júnior

josefisioexer@gmail.com

Supplementary Information

Supplementary Figure S1. Maximal carrying load per week over the course of 10 weeks of the training program of RET mice (red line, n = 14). Data are the mean ± SEM.

Supplementary Figure S2. Glucose tolerance and insulin sensitivity in resistance-trained mice immediately and 24h post-exercise. Blood glucose during ipGTT **(a)** and area under the curve (AUC) of total blood glucose concentration during ipGTT **(b)**, of CON (black bar, n = 7) and RET (red bar, n = 7), immediately post-exercise. Constant of glucose disappearance expressed by K_{ITT}, CON (black bar, n= 5) and RET (red bar, n = 4), immediately post-exercise **(c)**. Blood glucose during ipGTT **(d)** and area under the curve (AUC) of total blood glucose concentration during ipGTT **(e)**, of CON (black bar, n = 7) and RET (red bar, n = 7), 24h post-exercise. Constant of glucose disappearance expressed by K_{ITT}, CON (black bar, n= 5) and RET (red bar, n = 4), 24h post-exercise **(f)**. Data are the mean ± SEM. *$P \leq 0.05$ (Student's t-test).

Supplementary Figure S1.

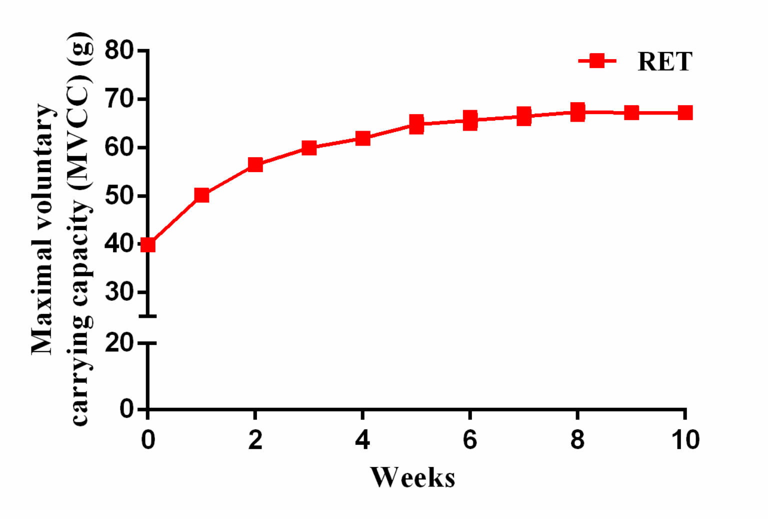

Supplementary Figure S2.

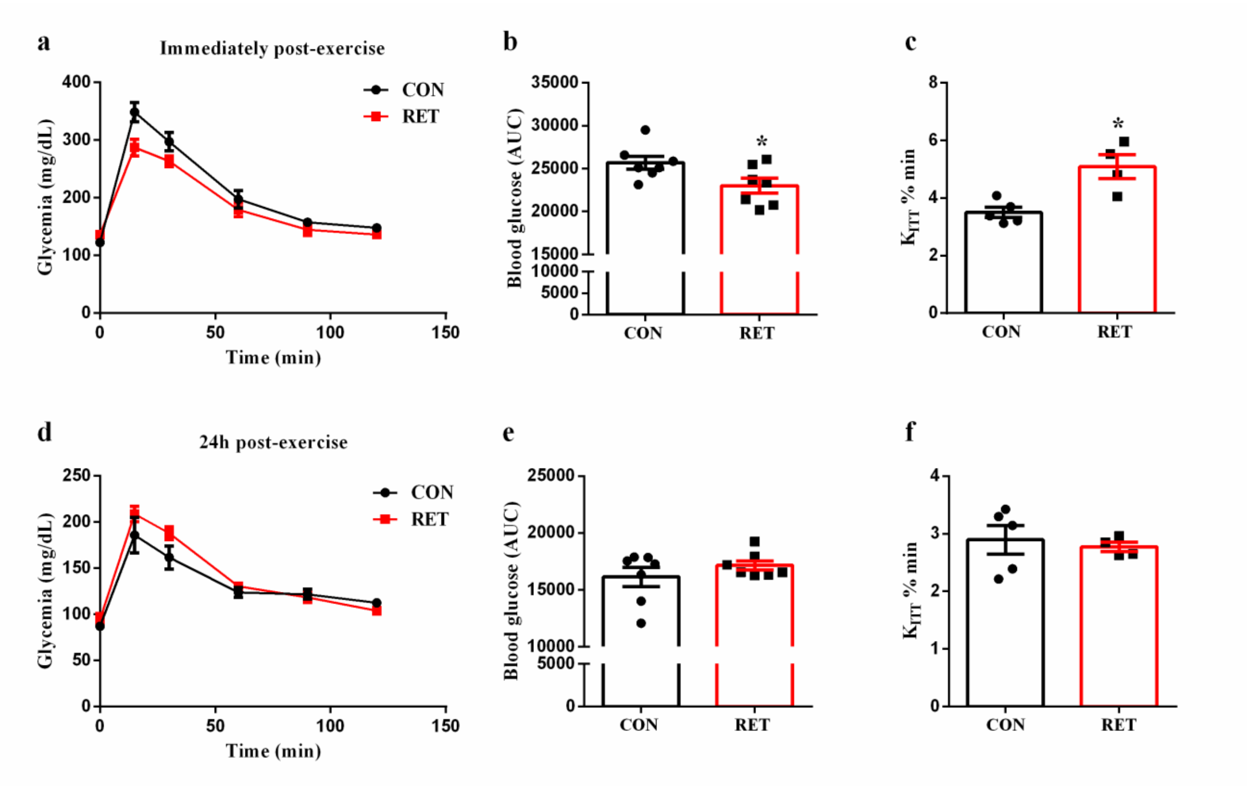

Supplement: Supplementary file 1 — Supplementary Information [file 41598_2021_88105_MOESM1_ESM.pdf]
